# Association between Meniere’s disease and air pollution in South Korea

**DOI:** 10.1038/s41598-021-92355-0

**Published:** 2021-06-23

**Authors:** Dong-Han Lee, Jiyeon Han, Myoung-jin Jang, Myung-Whan Suh, Jun Ho Lee, Seung Ha Oh, Moo Kyun Park

**Affiliations:** 1 Department of Otorhinolaryngology-Head and Neck Surgery, Konkuk University Medical Center, Seoul, Republic of Korea; 2 Medical Research Collaborating Center, Seoul National University Hospital, Seoul, South Korea; 3 Department of Otorhinolaryngology-Head and Neck Surgery, Seoul National University College of Medicine, Seoul National University Hospital, 101 Daehak-ro Jongno-gu, Seoul, 03080 Republic of Korea; 4 Sensory Organ Research Institute, Seoul National University Medical Research Center, 101 Daehak-ro Jongno-gu, Seoul, 03080 Republic of Korea

**Keywords:** Environmental sciences, Diseases, Risk factors

## Abstract

Meniere’s disease is thought to be a disorder of the inner ear function, affected by genetic and environmental factors. Several recent studies have shown that air pollution could affect middle and inner ear diseases. The purpose of this study was to investigate the relationship between the Meniere’s disease occurrence and air pollution status in Korea. This study used a time-stratified case-crossover design. Hospital visit data by Meniere’s disease were collected from the Korea National Health Insurance Service-National Sample Cohort (NHIS-NSC) database. Daily air pollution data for sulfur dioxide (SO_2_), nitrogen dioxide (NO_2_), carbon monoxide (CO), ozone (O_3_), and particulate matter (PM_10_: ≤ 10 μm in diameter, and PM_2.5_: ≤ 2.5 μm in diameter) were collected from the National Ambient air quality Monitoring Information System (NAMIS) database. We used two-stage analysis to assess the association between degree of air pollution and the occurrence of Meniere’s disease. In the first stage, region-specific analysis was conducted to estimate the odds ratios (ORs) of Meniere’s disease risk associated with each air pollutant exposure by using conditional logistic regression for matched case–control sets in 16 regions. In the second stage, region-specific ORs from the first stage were combined and the pooled effect estimates were derived through fixed and random effect meta-analysis. Subgroup analysis was conducted for age, sex, seasonality, and urbanization of residence. In total, 29,646 (32.1% males and 67.9% females) Meniere’s disease cases were identified from Korea NHIS-NSC database between 2008 and 2015. Overall, SO_2_, NO_2_, CO, and PM_10_ showed significant correlation with Meniere’s disease risk at immediate lags, and weaker correlation at delayed lags, whereas O_3_ showed slightly negative correlation at the immediate lag (lag0) and PM_2.5_ did not show strong correlation (SO_2_: 1.04 [95% confidence interval: 1.01, 1.06]; NO_2_: 1.08 [1.06, 1.11]; CO: 1.04 [1.02, 1.06]; O_3_: 0.96 [0.93, 0.99]: statistically significant ORs at lag0 are listed). These positive and negative associations between Meniere’s disease and each air pollutant were generally stronger in the age of 40–64, female, summer (June–August) season, and urban subgroups. Our results showed that hospital visits for Meniere’s disease were associated with the measured concentrations of ambient air pollutants SO_2_, NO_2_, CO, and PM_10_. Further studies are required to confirm these associations and find their mechanisms.

## Introduction

Meniere’s disease is a chronic illness of the inner ear characterized by episodic vertigo, fluctuating sensorineural hearing loss, ear fullness, and tinnitus, all of which decrease quality of life and could lead to permanent hearing loss. The annual incidence of Meniere’s disease was estimated at around 8–400 per 100,000 in the early 2000s^1,2^, and a recent study conducted in Korea reported similarly high values and a steep increase from 30.02 (in 2013) to 118.48 (in 2017) per 100,000^3^. According to a recent study in the UK, the annual health care costs associated with Meniere’s disease were estimated between USD 829.9 and 934.2 million, equating to $5112–$5748 per person per year^4^.

Since it was first described in 1861^5^, Meniere’s disease has been believed to be caused by the over-accumulation of endolymphatic fluid in the membranous labyrinth and subsequent membrane rupture^6,7^. Although several etiologic factors such as anatomical and vestibular abnormalities, allergy, auto-immune disease, viral infection, and trauma are thought to be associated with Meniere’s disease, its exact pathophysiology remains unclear^8–10^.

Diet, stress, and lifestyle are associated with the progress of Meniere’s disease, and environmental factors such as atmospheric pressure and humidity are strongly related to the aggravation of Meniere’s symptoms^11^, but the relationship between this disease and environmental pollution is not yet well known. Several recent studies have shown that environmental pollution affects middle and inner ear diseases^12–15^. Therefore, identifying the relationship between air pollution and Meniere’s disease can help understand the pathophysiology of the disease and manage it.

The Korea National Health Insurance database provides health services with big data related to medical care, and we can integrate these data and air pollution data from the Korea Environment Corporation to analyze the association between the frequency of Meniere’s disease hospital visits and the level of environmental pollution. The purpose of this study was to investigate the relationship between the Meniere’s disease and air pollution.

## Patients and methods

### Database and study population

This study used data from the Korea National Health Insurance Service-National Sample Cohort (NHIS-NSC) database. All Korean people are given a unique registration number from birth and are obliged to join the NHIS, and all Korean hospitals use this identification number, so medical insurance claim records do not overlap and are not missing. The NHIS selected about 2% (1 million) of samples from the entire Korean population (50 million) and a previous study verified that these samples represented the entire population appropriately^16^. For each participant, this database contains comprehensive healthcare information including (1) patient information (sex, age, area of residence, and income level), (2) medical insurance claim codes (treatment procedures and prescriptions), (3) diagnostic codes in the format of the International Classification of Diseases-10 (ICD-10), (4) medical examination data, and (5) death records.

### Study design

This study used a time-stratified case-crossover design, which was developed to study the effect of transient exposures on acute-onset events^17,18^. In the case-crossover design, each case’s exposure prior to the event is compared to exposure in referent periods. In this way, each case serves as his or her own control, and time-invariant factors such as sex, ethnicity, and genetic background are automatically controlled. Furthermore, infrequently changing variables such as underlying chronic disease, socioeconomic status, area of residence, occupation, marriage status, seasonality, and age are also controlled by choosing control days close enough to the case day^17,19–22^.

Our hypothesis was that air pollution could trigger the onset of Meniere’s disease. We selected the day of the insurance claim due to Meniere’s disease as the case day. To avoid bias due to temporal variation or trend in exposures and to control for patterns in health care use depending on the day of the week, we selected control days in the same month and the same day of the week as the case day. Therefore, each case day had 3 or 4 control days for comparison. For example, if a subject visited the hospital due to Meniere’s disease on a Monday in June 2010, all other Mondays in June 2010 were chosen as the control days, and the air pollution levels of the case day and control days were compared^20^.

This study was approved by the Seoul National University Hospital (SNUH) Institutional Review Board (IRB) (IRB No. 1806-001-948). All analyses were performed in accordance with relevant guidelines and regulations of the Ethics Committee of Seoul National University Hospital. Written informed consent was waived by the Institutional Review Board.

### Participant selection and variables

In the NHIS-NSC data from February 2008 to December 2015, we traced a total of 882,230 individuals aged 19–79 to select subjects who visited hospitals (including emergency room visits and hospital admissions) for Meniere’s disease (ICD-10 code H81.0) as the first-time diagnosis (without previous diagnosis with the same code during at least 1 year), to exclude regular follow-up visits without Meniere attack symptoms. From the selected participants, we collected the data on sex, age, date of hospital visit, and residence.

### Air pollution data

Since April 2004, the Korea Environment Corporation has established the National Ambient air quality Monitoring Information System (NAMIS) to collect and manage air pollution data such as the levels of sulfur dioxide (SO_2_), nitrogen dioxide (NO_2_), carbon monoxide (CO), ozone (O_3_), and particulate matter (PM) measured in air pollution measurement networks nationwide. Real-time concentrations of air pollutants are measured by automatic monitoring devices at 398 monitoring spots located in 112 areas (as of December 2018) all over the country. The NAMIS collects these data and provides them to the general public online (http://www.airkorea.or.kr). We used this database between January 1, 2008 and December 31, 2015.

We selected six pollutants that attract wide interest: (1) SO_2_ (measured by a pulse ultraviolet fluorescence method), (2) NO_2_ (by chemiluminescence), (3) CO (by a non-dispersive infrared method), (4) O_3_ (by an ultraviolet photometric method), (5) PM_10_ (PM ≤ 10 μm in diameter; by a ray absorption method), and (6) PM_2.5_ (PM ≤ 2.5 μm in diameter; by a ray absorption method). However, the data of PM_2.5_ were available only from 2015. For analysis, pollution information was classified into 16 regions on the basis of administrative districts.

### Statistical analysis

The association between air pollution and the occurrence of Meniere’s disease was examined via a two-stage analysis. In the first stage, the association in each region was estimated with the odds ratios (ORs) and 95% confidence intervals (CIs) by using conditional logistic regression for matched case–control sets. In this analysis, the exposure to each air pollutant was fitted to a conditional logistic regression model using both the single lag structure (lag 0, exposure on the case or control day; lag 1, 2, …, and 7; exposure 1, 2, …, and 7 days prior to the case or control day) and cumulative lag structure (lag 0–1, lag 0–2, …, and lag 0–7; the moving average of exposure between the case or control day [0] and respective days prior to day 0) to consider the immediate, delayed, and cumulative effects of air pollution^20^. We analyzed the data using these various lag structures because we assumed that there may be a time interval between exposure to an air pollutant, the occurrence of Meniere’s disease, and subsequent hospital visits. We included meteorological variables (temperature, rainfall, wind speed, and relative humidity) in the conditional logistic regression analysis; those variables were adjusted for the 4 day moving average (lag 0–3) with a regression spline of 3 degrees and 4 knots for temperature to control for a possible non-linear association and with a linear term for the other variables^20,23,24^. All meteorological data were obtained from the Korea Meteorological Administration.

#### Subgroup analysis

We conducted region-specific subgroup analyses, which were stratified by potential effect modifiers such as age (three age groups: 19–39, 40–64, and 65–79 years), sex, and season (spring: March–May; summer: June–August, fall: September–November; winter: December–February) to find out the differences in Meniere’s disease risk according to subgroup classification.

#### Joint-effect analysis

In addition, region-specific joint (combined) effects of multiple pollutants were analyzed by including multiple pollutants in the conditional logistic model in the following combinations: traffic gases (NO_2_, CO), oxidant gases (SO_2_, NO_2_, O_3_), and criteria pollutants (SO_2_, NO_2_, CO, O_3_, PM_10_)^25^. Following the method of Winquist et al.^25^ a model including only individual pollutants of these combinations without interaction terms (non-interaction model) and a model including linear interaction terms were analyzed and compared. In the interaction model, the joint effects of the concentration change corresponding to the interquartile range (IQR) of each pollutant were calculated, starting at the 15th, 25th, and 35th percentile levels of each pollutant.

In the region-specific analysis, regions with small numbers of cases (< 20) were excluded because we failed to obtain results from data with a small number of cases in multivariable logistic models that included air pollutants and meteorological variables due to model convergence problem. In the analysis of association between PM_2.5_ and Meniere’s disease, where only 2015 cases were used, one region (Chungnam) was excluded. In the subgroup analysis, six regions (Daejeon, Ulsan, Jeonbuk, Jeju, Chungnam, and Chungbuk) were excluded.

In the second stage, region-specific ORs from the first stage were combined and the pooled effect estimates were derived through fixed- and random-effect meta-analysis, which provided the overall effect of each pollutant on Meniere’s disease throughout the regions as well as the regional variation of that effect. The results of the fixed-effect model were accepted when heterogeneity I^2^ was lower than 50%, and the results of the random-effect model were accepted when I^2^ was higher than 50%^26^. Additionally, pooled effect estimates were derived separately according to the grouping by urbanization of residence (urban or rural) to find out whether the occurrence of Meniere’s disease is affected by urban and rural residence. Sixteen regions were classified into urban areas (1 special and 6 metropolitan cities: Seoul, Busan, Daegu, Incheon, Gwangju, Daejeon, and Ulsan) and rural areas (9 provinces: Gyeonggi, Ganwon, Chungbuk, Chungnam, Jeonbuk, Jeonnam, Gyeongbuk, Gyongnam, and Jeju) on the basis of administrative divisions.

All statistical analyses were conducted using SAS Enterprise Guide^®^ software (version 7.13; SAS Institute, Cary, NC, USA) and R: A Language and Environment for Statistical Computing (version 3.3.3; R Core Team, Vienna, Austria). All results were presented as estimated ORs and 95% CIs per IQR increase at each air pollutant concentration^20^. IQR for each air pollutant was calculated from its 24-h average concentration in all regions as follows: 2.86 ppb for SO_2_, 12.52 ppb for NO_2_, 0.1 ppm for CO, 16.3 ppb for O_3_, 27.19 μg/m^3^ for PM_10_, and 16.83 μg/m^3^ for PM_2.5_.

## Results

### Patient and air pollution distribution

In total, 29,646 (32.1% males and 67.9% females) Meniere’s disease cases were identified from Korea NHIS-NSC database between 2008 and 2015; 4464 (33.6% males and 66.4% females) cases were included in 2015 in the analysis of association with PM_2.5_, and 3972 (33.6% males and 66.4% females) were included in the subgroup analysis of association with PM_2.5_ (Table 1). Among the three age groups, 40–64 years was the largest (49.9% in 2008 to 2015, 51.8% in 2015). The distribution of Meniere’s disease did not differ significantly among seasons, but it occurred the least frequently in winter (23.1% in 2008 to 2015, 18.1% in 2015). The number of cases of Meniere’s disease by region was from 438 (1.5%) in Jeju to 5756 (19.4%) in Gyeonggi (Table 2). Supplementary Table S1 shows the difference in air pollution levels and meteorological variables between the case and the control days at various lags. Except for O_3_, the concentrations of air pollutants were slightly higher on case days than on control days for most time delays, but no such tendency was found for most of meteorological variables. Supplementary Figure S1 shows weekly concentration of each air pollutant in a typical year (2015) when all pollutants were analyzed. There was noticeable concentration variability between adjacent weeks for all pollutants.

**Table 1:** Characteristics of 29,646 Meniere’s disease cases according to subgroups in Korea between 2008 and 2015.

Study period:	2008–2015		2015	
Analysis:	SO_2_, NO_2_, CO, O_3_, PM_10_		PM_2.5_^a^	
Total	29,646 (n)	100 (%)	3972 (n)	100 (%)
**Age**				
19–39	6161	20.8	830	20.9
40–64	14,791	49.9	2058	51.8
65–79	8694	29.3	1084	27.3
**Season**^b^				
Spring	7585	25.6	1119	28.2
Summer	7443	25.1	1043	26.3
Fall	7769	26.2	1091	27.5
Winter	6849	23.1	719	18.1
**Sex**				
Male	9519	32.1	1334	33.6
Female	20,127	67.9	2638	66.4

^a^In subgroup analysis for PM_2.5_, regions (Daejeon, Ulsan, Jeonbuk, Jeju, Chungnam, and Chungbuk) with fewer than 20 cases in the subgroup were excluded.

^b^Definitions for seasons are as follows: Spring (March–May), Summer (June–August), Fall (September–November), and Winter (December–February).

**Table 2:** Regional Meniere’s disease cases and air pollution distribution in Korea between 2008 and 2015.

Study period:	2008–2015			2008–2015			2008–2015			2008–2015			2008–2015			2008–2015			2015^a^			2015^a^
Region	Cases of MD	Sample population	Incidence per 1000	SO_2_ percentiles (ppb)			NO_2_ percentiles (ppb)			CO percentiles (0.1 ppm)			O_3_ percentiles (ppb)			PM_10_ percentiles (μg/m^3^)			PM_2.5_ percentiles ( μg/m^3^)			Cases of MD
	(n)	(n)	(n)	10	50	90	10	50	90	10	50	90	10	50	90	10	50	90	10	50	90	(n)
Gangwon	1280	33,008	38.78	1.6	2.9	6.9	8.1	13.0	21.6	3.2	4.9	8.5	17.1	27.2	45.3	20.4	41.4	76.0	10.5	22.3	45.7	241
Gyeonggi	5756	245,245	23.47	3.2	4.9	7.9	16.2	27.3	44.3	3.7	5.2	8.5	9.2	21.3	38.2	25.4	49.9	89.3	9.8	22.2	45.2	1012
Gyeongnam	2080	66,927	31.08	2.6	4.0	6.3	9.6	16.1	28.0	2.8	4.0	6.1	15.4	27.8	43.2	25.0	42.0	72.7	12.5	24.0	38.7	345
Gyeongbuk	1426	56,536	25.22	2.9	4.3	6.8	8.5	13.3	21.8	3.4	4.8	7.4	17.1	27.9	44.7	25.0	41.9	73.9	11.0	19.5	38.5	234
Gwangju	1408	29,451	47.81	2.3	3.4	5.4	10.4	18.3	31.3	3.3	4.7	7.4	12.3	25.6	42.1	19.0	38.0	72.0	10.8	23.4	42.5	207
Daegu	1299	50,343	25.80	2.3	3.9	7.6	11.7	21.1	38.6	2.9	4.5	7.3	9.4	23.3	41.9	23.3	42.5	77.0	10.5	22.4	40.1	232
Daejeon	923	31,799	29.03	1.8	3.4	6.7	10.8	18.9	33.3	2.9	4.4	7.9	8.6	22.3	40.3	17.9	38.4	71.3	11.0	25.0	50.0	57
Busan	1906	72,844	26.17	3.8	5.9	8.8	12.0	19.4	31.2	2.7	3.8	5.4	15.4	27.3	40.5	26.5	42.1	74.6	12.1	23.5	41.1	303
Seoul	5186	222,083	23.35	3.4	4.9	7.8	18.7	31.7	51.2	3.4	4.9	8.5	7.2	19.8	36.3	19.4	42.0	79.3	9.9	20.8	38.8	819
Ulsan	454	23,093	19.66	4.6	7.0	12.4	13.1	21.8	34.5	3.3	4.8	6.9	14.2	24.7	39.1	24.4	42.3	77.7	10.2	22.0	42.5	70
Incheon	1470	59,747	24.60	4.2	6.1	9.6	13.6	23.7	40.7	3.6	5.2	8.6	12.8	25.6	41.3	24.9	46.3	84.3	11.9	24.9	49.5	261
Jeonnam	2233	39,842	56.05	3.9	5.7	8.9	9.2	14.5	23.7	3.3	4.6	6.5	17.0	29.6	43.4	21.4	35.4	63.3	11.8	22.7	39.7	318
Jeonbuk	1550	37,405	41.44	2.9	4.0	5.9	8.5	13.4	22.4	3.2	4.4	6.8	13.5	25.2	41.3	23.5	45.3	80.5	12.0	31.2	60.0	172
Jeju	438	11,546	37.94	1.3	2.0	4.0	4.5	8.5	14.3	2.3	3.5	5.5	23.5	37.8	52.5	21.0	36.0	71.3	9.5	20.5	38.8	68
Chungnam	1281	45,833	27.95	2.3	3.7	6.2	8.6	14.1	23.6	3.4	4.8	7.7	15.7	28.0	43.7	20.6	40.5	73.6	11.0	23.0	44.0	
Chungbuk	956	32,644	29.29	2.0	3.8	8.5	11.0	18.7	32.5	2.8	4.8	9.8	9.8	22.9	42.5	22.4	49.5	89.5	7.0	22.5	51.8	125
All regions	29,646																					4464

*MD* Meniere’s disease.

^a^In analysis for PM_2.5_, Chungnam region with fewer than 20 cases was excluded.

### Association between air pollution and Meniere’s disease

**Figure 1:**
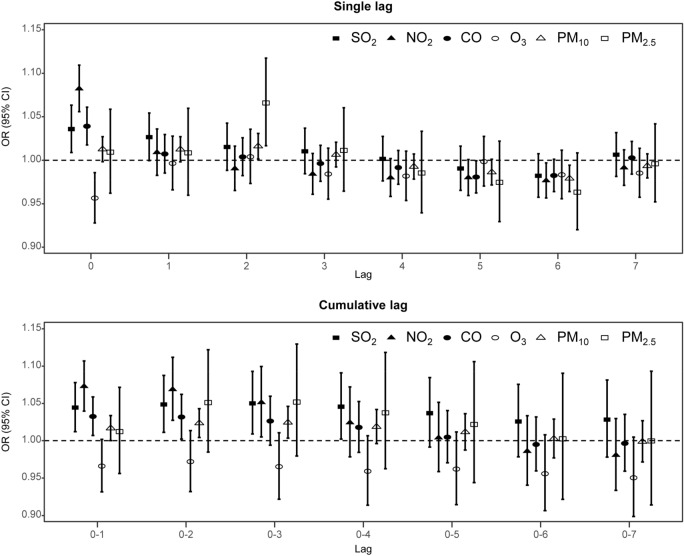
Association between Meniere’s disease and air pollution. The pooled effect estimates of odd ratios (ORs) and 95% CIs for Meniere’s disease hospital visits considering interquartile range increase in each air pollutant concentration are presented according to various lag structures to take into account the immediate, delayed, and cumulative effects of air pollutants. Single lag indicates exposure on the case or control day (lag0) or on day 1–7 prior to the case or control day (lag1–lag7). Cumulative lag indicates the moving average of exposure between the case or control day and ‘n’ days prior to the case or control day (lag0–n). This figure was drawn using R: a Language and Environment for Statistical Computing (version 3.3.3; R Core Team, Vienna, Austria, https://www.R-project.org).

The final results of two-staged analysis are summarized in Fig. 1, which shows the pooled effect estimates that combine 16 region-specific estimates of Meniere’s disease risk associated with an increase in IQR of each air pollutant according to various lag structures. Overall, SO_2_, NO_2_, CO, and PM_10_ showed significant correlation with Meniere’s disease risk at immediate lags, and weak correlation at delayed lags. O_3_ showed a slightly negative correlation at immediate lags, whereas PM_2.5_ showed no strong correlation. The estimated pooled effect ORs at lag0 were as follows: SO_2_: 1.04 (95% CI 1.01, 1.06); NO_2_: 1.08 (1.06, 1.11); CO: 1.04 (1.02, 1.06); O_3_: 0.96 (0.93, 0.99) (Supplementary Figure S2).

### Analysis according to subgroups and potential effect modifiers

**Figure 2:**
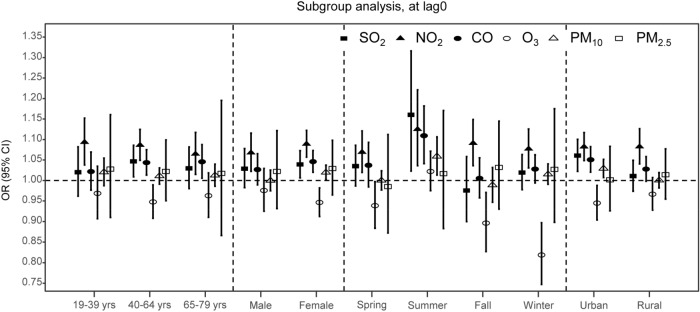
Subgroup analysis of association between Meniere’s disease and air pollution. The pooled effect estimates of odd ratios (ORs) and 95% CIs for Meniere’s disease hospital visits considering interquartile range increase in each air pollutant concentration are presented according to subgroup (potential effect modifiers: age, sex, season, and urbanization of residence). This figure was drawn using R: a Language and Environment for Statistical Computing (version 3.3.3; R Core Team, Vienna, Austria, https://www.R-project.org).

Figure 2 shows subgroup-specific estimated effects according to three age subgroups, sex, four seasons (spring, summer, fall, and winter), and urbanization of residence (urban or rural). Among the age subgroups, the correlation between air pollution and Meniere’s disease risk was strongest at the age of 40–64, especially for NO_2_, SO_2_, and CO. O_3_ showed a generally negative correlation in all age subgroups, and PM_10_ and PM_2.5_ showed no significant correlation in any age subgroup. Statistically significant results at lag0 were as follows: SO_2_: 1.05 (95% CI 1.01, 1.09) at age 40–64; NO_2_: 1.09 (1.04, 1.15) at age 19–39, 1.09 (1.05, 1.12) at age 40–64, 1.06 (1.01, 1.12) at age 65–79; CO: 1.04 (1.01, 1.07) at age 40–64, 1.05 (1.01, 1.09) at age 65–79; O_3_: 0.95 (0.91, 0.99) at age 40–64.

These positive and negative associations between each air pollutant and Meniere’s disease were more significant in women than in men in sex subgroup analysis, and PM_10_ in women (but not in men) showed significant association with Meniere disease risk at some lags. Statistically significant results at lag0 were as follows: SO_2_: 1.04 (95% CI 1.01, 1.07) in females; NO_2_: 1.07 (1.02, 1.12) in males, 1.09 (1.06, 1.12) in females; CO: 1.05 (1.02, 1.07) in females; O_3_: 0.95 (0.91, 0.98) in females.

Seasonal impact analysis showed a more significant correlation in summer than in the other seasons. Statistically significant results at lag0 were as follows: SO_2_: 1.13 (95% CI 1.05, 1.22) in summer; NO_2_: 1.07 (1.02, 1.12) in spring, 1.12 (1.04, 1.22) in summer, 1.09 (1.04, 1.15) in fall, 1.08 (1.03, 1.13) in winter; CO: 1.11 (1.04, 1.18) in summer; PM_10_: 1.06 (1.01, 1.11) in summer.

The effect of air pollution associated with Meniere’s disease was more significant in urban than in rural areas. SO_2_, NO_2_, CO, and PM_10_ at lag0, and O_3_ at lag0 showed significant negative correlation in urban areas. Statistically significant results at lag0 were as follows: SO_2_: 1.06 (95% CI 1.02, 1.10) in urban areas; NO_2_: 1.08 (1.05, 1.12) in urban areas, 1.08 (1.04, 1.13) in rural areas; CO: 1.05 (1.02, 1.08) in urban areas; O_3_: 0.94 (0.90, 0.99) in urban areas; PM_10_: 1.03 (1.01, 1.05) in urban areas.

### Joint-effect analysis

**Figure 3:**
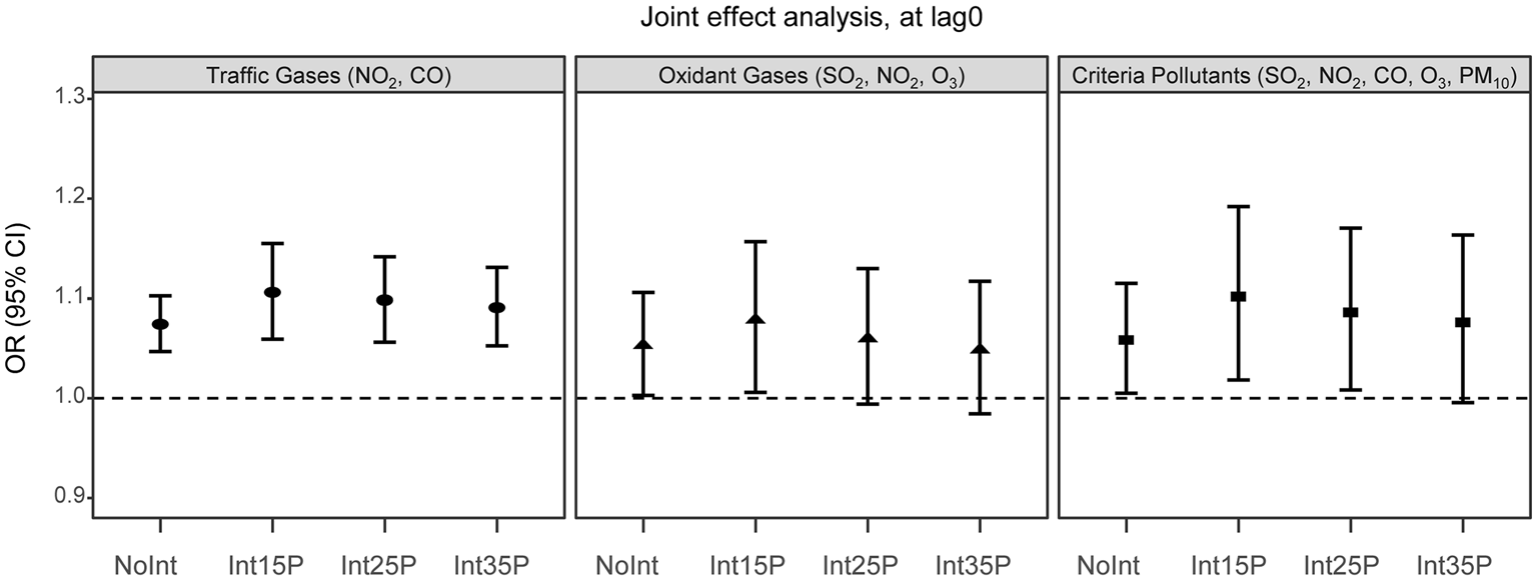
Joint effect of specific pollutant combinations for Meniere’s disease. Joint effect estimates of odd ratios (ORs) and 95% CIs for Meniere’s disease hospital visits considering interquartile range (IQR) increase in concentrations of specific pollutant combinations on the case or control day (lag0) are presented using conditional logistic model including multiple pollutants with and without interactions. In the interaction models, the joint effects for the concentration change corresponding to the IQR were calculated, starting at the 15th, 25th, and 35th percentile level of each pollutant. This figure was drawn using R: a Language and Environment for Statistical Computing (version 3.3.3; R Core Team, Vienna, Austria, https://www.R-project.org). NoInt: No interactions; Int15P: interaction, starting at 15th percentile; Int25P: interaction, starting at 25th percentile; Int35P: interaction, starting at 35th percentile.

Joint-effect analysis showed that increases in the concentrations of all pollutant combinations investigated were associated with increases in Meniere’s disease hospital visits (Fig. 3). The pooled estimated ORs at lag0 for the joint effect by an IQR increase in the non-interaction model were as follows: traffic gases (NO_2,_ CO): 1.07 (95% CI 1.05, 1.10) and criteria pollutants (SO_2_, NO_2_, CO, O_3_, PM_10_): 1.06 (1.01, 1.11). The joint-effect estimates in the interaction model were as follows: traffic gases: 1.11 (95% CI 1.06, 1.15) when starting at the 15th percentile, 1.10 (1.06, 1.14) when starting at the 25th percentile, 1.09 (1.05, 1.13) when starting at the 35th percentile; oxidant gases (SO_2_, NO_2_, O_3_): 1.08 (1.01, 1.16) when starting at the 15th percentile; criteria pollutants: 1.10 (1.02, 1.19) when starting at the 15th percentile, 1.09 (1.01, 1.17) when starting at the 25th percentile. Compared to the non-interaction model, the estimated joint effects of the interaction model were slightly larger or similar.

## Discussion

Meniere’s disease is generally hypothesized to be caused by endolymphatic hydrops, but there are still debates on the exact pathophysiologic mechanism^27^. Although endolymphatic hydrops is identified in most Meniere patients, animal models with induced endolymphatic hydrops have failed to develop the characteristic attacks with Meniere-like symptoms^28–30^. In addition, there are patients with endolymphatic hydrops without Meniere symptoms^31,32^. Conversely, there are patients with Meniere symptoms but without any sign of endolymphatic hydrops in temporal bone histopathology^31,32^. Anatomical variances such as short and narrow vestibular aqueduct found in Meniere’s patients are thought to cause endolymphatic hydrops^33–35^. The elevated serum levels of immune mediators in Meniere patients suggest that Meniere’s disease may be related to allergies or immunologic factors^36–46^. Clinical features and its course, MR imaging, and serological and epidemiologic evidence support the viral etiology of Meniere’s disease, and histopathologic analysis also suggests viral infection as a causative factor for structural abnormalities of the endolymphatic duct and sac^47–52^.

Extensive efforts have been made to find an effective management and treatment for Meniere’s disease, and to understand its pathophysiologic mechanism. Traditional treatment strategies for Meniere’s disease include life style modification (e.g., stress relief, getting enough sleep, and avoiding alcohol and smoking)^53^, diet therapy (e.g., low salt diet^54^, high water intake^53,54^, and limitation of caffeine), medication (e.g., diuretics^54–57^, betahistine^55,58–60^, intratympanic corticosteroids^9,61–63^, and intratympanic gentamycin^64,65^), and surgery (e.g., ventilation tube insertion with or without middle ear pressure application^66–69^, endolymphatic sac decompression^70^, and although destructive, vestibular neurectomy or labyrinthectomy in selected intractable cases^71^). However, due to the lack of well-designed randomized controlled trials, the evidence for these treatment strategies is still weak.

Several studies have suggested that environmental pollution could be associated with inner ear problems^72^. In 2012, studies that analyzed the data from the U.S. National Health and Nutritional Examination Survey (NHANES) showed that exposure to cadmium and lead might be associated with hearing loss^73^ and vestibular dysfunction^74^. Some studies have suggested an association between environmental pollution and middle ear disease such as otitis media^75–77^. Therefore, we hypothesized that Meniere’s disease, which is believed to be caused by physiological dysfunction of the inner ear, may also be affected by environmental pollution. In 2017, Han and colleagues analyzed the NHIS-NSC data and reported that hospital visits due to Meniere’s disease were associated with environmental PM exposure, especially in the elderly^12^. However, among various air pollutants, this study analyzed only PM_10_ and PM_2.5_ in only one city, Seoul. Similar to this study, we also used time-stratified case-crossover analysis, which is thought to be an effective epidemiologic tool for assessing the triggering factor of a disease, but we included many more pollutants and regions in the analysis.

Our analysis suggests a possible association between the onset of Meniere’s disease and concentration of air pollutants such as SO_2_, NO_2_, and CO, as well as PM_10_ and PM_2.5_. Our joint-effect analysis also shows that specific air pollutant mixtures such as traffic gases (NO_2_, CO), oxidant gases (SO_2_, NO_2_, O_3_), and criteria pollutants (SO_2_, NO_2_, CO, O_3_, PM_10_) may be associated with the onset of Meniere’s disease. Air pollution can induce allergic reaction or systemic inflammation^78^, and this inflammation could affect the inner ear physiology. On the other hand, inflammation caused by air pollution can cause psychological distress symptoms^79^. In 2004, Söderman et al. reported that prior emotional stress could affect the Meniere’s attack^80^. In 2012, Lim et al. reported that air pollutants such as PM_10_, NO_2_, and O_3_ may increase depressive symptoms in the elderly^81^. Many other reports show the association between air pollution and headache and migraine symptoms, which are commonly co-occurring symptoms in Meniere patients^82–84^. These findings may be related to each other and provide a clue to how air pollution causes Meniere’s disease.

Our subgroup analysis showed that the correlation between air pollution and Meniere’s disease risk was strongest in the age of 40–64, female, and summer (June–August) season subgroups. Meniere’s disease is known to be more common in middle-aged adults than in children or the elderly^85^, and more common in women than in men^2,86^. In 2017, Schmidt et al. reported that weather conditions such as low atmospheric pressure and high humidity are associated with Meniere’s attacks^11^. Therefore, a possible interpretation of this result is that air pollution may have a synergetic effect in these subgroups. We found a more significant association in urban than in rural areas, but this association was weaker than we expected. As in a previous study conducted in Korea^15^, we grouped 16 regions into urban and rural on the basis of administrative divisions, but the concentration of pollutants in urban areas was not necessarily higher than in rural areas, probably because industrial facilities or plants are distributed in some rural areas.

Interestingly, we observed a generally negative association between O_3_ level and onset of Meniere’s disease, although ozone is a toxic gas and no beneficial effects of ground-level ozone on human health are known. A possible interpretation is that this negative association is partially due to a coincidental negative correlation of O_3_ with other pollutants. In our study, Spearman correlation coefficient Rho was − 0.18 for O_3_ and SO_2_, − 0.29 for O_3_ and CO, and − 0.40 for O_3_ and NO_2_. Previous studies have also reported negative correlation between O_3_ and other pollutants^78,87–89^. In a study that assessed the association between air pollution and suicide risk, O_3_ levels were less associated with suicide risk than those of other pollutants such as SO_2_, NO_2_, CO, and PM_10_^20^. However, there is a lack of evidence that O_3_ is always negatively correlated with other pollutants. CO, NO_2_, and PM are produced mainly by automobiles, and SO_2_ is produced by combustion of coal and sulfur-containing fossil fuels, but ground-level O_3_ is typically produced from nitrogen oxides and volatile organic compounds by photochemical reactions that require heat and sunlight, so the O_3_ level is high in summer^90^. Another possible interpretation is that this negative correlation may be due to reduced outdoor activity in extreme weather conditions, when high temperatures and sunlight result in high O_3_ levels^20^. At this moment, it is unclear whether O_3_ has a direct impact on Meniere’s disease.

Contrary to our expectation, we found no significant association between PM_2.5_ and Meniere’s disease, possible because of insufficient sample size for PM_2.5_. The data for PM_2.5_ were available only for 2015, and many regions were excluded from the subgroup analysis due to a statistical processing problem. Since various factors may affect the onset of Meniere’s disease, we think that it is difficult to demonstrate the effects of ambient air pollutants that are present in small amounts in the atmosphere if we do not have enough samples.

Several limitations should be considered in the interpretation of the results of this study. The data on the occurrence of Meniere’s disease we collected might differ from reality. The diagnosis of Meniere’s disease was not confirmed pathologically in clinical settings and was based on clinical symptoms. The diagnostic criteria of individual clinicians might not be identical, and because the data on the occurrence of Meniere’s disease were based on the insurance claim code, the occurrence might have been underestimated, which is a fundamental limitation of this type of research. In addition, there could be a time difference between exposure to air pollution, the occurrence of Meniere’s disease, and the resulting hospital visits, so we analyzed the data using various lag structures, but there might be a lag heterogeneity for each individual. However, we believe that the delay between the patient’s symptoms and the visit to the hospital would not have been very long. To collect the data on the first or acute attack of Meniere’s disease and exclude regular visits, we selected patients who had not been diagnosed with the same code in the previous year, and included emergency room visits and hospital admissions. Generally, the more pronounced the discomfort symptoms are, the more likely the hospital visit will be made immediately rather than delayed. If a clinician made a diagnosis under the exact insurance claim code of Meniere’s disease, we assume that the symptoms would have been clear enough for diagnosis. Korea is relatively small in terms of land area, and most of the population is concentrated in urban areas, so patients have good geographical access to hospitals. As the NHIS covers the entire Korean population and medical fees are low, the threshold for the use of medical care is also low^91^. Given that, in the majority of cases it can be assumed that the patient’s visit to the hospital was not considerably delayed. Another limitation is that air pollution was measured at specific locations in each city and the average was taken, and since our study did not take into account the actual time of the individual’s outdoor activities during the day, the individual’s actual pollutant exposure might not be precisely reflected. Also, several confounding factors such as sleep quality, stress, diet, and symptom severity were not considered in the analysis because of to the lack of data. In addition, our interpretations may be applicable only to the Korean region because inter-regional differences in environmental conditions and ethnicities may affect the results of analysis.

Although the interpretation of our results may be controversial, we have identified a possible association between air pollution and the occurrence of Meniere’s disease. But the mechanism by which air pollution affects Meniere disease is still unclear. Therefore, further animal study or well controlled prospective study is needed to support our interpretations of the data.

## Conclusion

Our time-stratified case-crossover analysis showed that Meniere’s disease hospital visits were associated with the measured concentrations of ambient air pollutants such as SO_2_, NO_2_, CO, and PM_10_. This association was stronger in the age of 40–64, female, summer (June–August) season, and urban subgroups. Further studies are needed to confirm these associations and determine their mechanisms.

## Supplementary Information

Supplementary Information 1.

## Data Availability

All the data used in this study are available from the Korea National Health Insurance Service-National Sample Cohort (NHIS-NSC) database, Korea National Ambient air quality Monitoring Information System (NAMIS) database, and the Korea Meteorological Administration database.
