# Supplementary material for: Association between Meniere’s disease and air pollution in South Korea
Source: Sci Rep. 2021 Jun 23;11:13128. doi: 10.1038/s41598-021-92355-0 (PMC8222348; doi:10.1038/s41598-021-92355-0)
Supplement: Supplementary file 1 — Supplementary Information 1. [file 41598_2021_92355_MOESM1_ESM.doc]

**Association** **between Meniere’s disease and air pollution in South Korea**

Dong-Han Lee, MD^1^, Jiyeon Han^2^, Myoung-jin Jang^2^, Myung-Whan Suh, MD, PhD^3,4^, Jun Ho Lee, MD, PhD^3,4^, Seung Ha Oh, MD, PhD^3,4^, Moo Kyun Park, MD, PhD^3,4*^

**Authors’ affiliations**:

*^1^Department of Otorhinolaryngology-Head and Neck Surgery, Konkuk University Medical Center, Seoul, Republic of Korea*

*^2^Medical Research Collaborating Center, Seoul National University Hospital, Seoul, South Korea*

*^3^Department of Otorhinolaryngology-Head and Neck Surgery, Seoul National University College of Medicine, Seoul National University Hospital, Seoul, South Korea*

*^4^Sensory Organ Research Institute, Seoul National University Medical Research Center, Seoul, South Korea*

**Correspondence to:** Moo Kyun Park, MD, PhD*

*Department of Otorhinolaryngology-Head and Neck Surgery, Seoul National University College of Medicine, Seoul National University Hospital, Seoul, South Korea*

*Sensory Organ Research Institute, Seoul National University Medical Research Center, Seoul, South Korea*

*101 Daehak-ro Jongno-gu, Seoul 03080, Republic of Korea*

*Tel: 82-2-2072-2448, Fax: 82-2-2072-2447*

*E-mail: entpmk@gmail.com*

**Supplementary Table 1. Air pollution levels and meteorological variables on case and control days for Meniere’s disease in Korea, between 2008 and 2015**

|  | Case day |  |  |  | Control day |  |  |  | Mean difference |  |  |  |
| --- | --- | --- | --- | --- | --- | --- | --- | --- | --- | --- | --- | --- |
|  | mean±SD | Q1 | Med | Q3 | mean±SD | Q1 | Med | Q3 | mean±SD | Q1 | Med | Q3 |
| SO_2_ (ppb) |  |  |  |  |  |  |  |  |  |  |  |  |
| lag0 | 5.0±2.2 | 3.5 | 4.6 | 6.1 | 5.0±2.2 | 3.5 | 4.6 | 6.1 | 0.03±2.16 | -1.0 | 0.0 | 1.0 |
| lag1 | 5.0±2.2 | 3.5 | 4.6 | 6.1 | 5.0±2.2 | 3.5 | 4.6 | 6.0 | 0.02±2.14 | -1.0 | 0.0 | 1.0 |
| lag2 | 5.0±2.2 | 3.5 | 4.6 | 6.1 | 5.0±2.2 | 3.5 | 4.6 | 6.0 | 0.02±2.11 | -1.0 | 0.0 | 1.0 |
| lag0-1 | 5.0±2.1 | 3.6 | 4.7 | 6.1 | 5.0±2.0 | 3.6 | 4.7 | 6.1 | 0.03±1.86 | -0.9 | 0.0 | 0.9 |
| lag0-2 | 5.0±2.0 | 3.6 | 4.7 | 6.0 | 5.0±1.9 | 3.6 | 4.7 | 6.0 | 0.02±1.64 | -0.8 | 0.0 | 0.8 |
| NO_2_ (ppb) |  |  |  |  |  |  |  |  |  |  |  |  |
| lag0 | 23.6±11.8 | 14.6 | 20.9 | 30.2 | 23.2±11.7 | 14.3 | 20.6 | 29.6 | 0.34±11.22 | -5.4 | 0.3 | 6.1 |
| lag1 | 22.6±11.5 | 13.8 | 19.9 | 29.0 | 22.5±11.5 | 13.8 | 19.8 | 28.8 | 0.11±11.03 | -5.6 | 0.1 | 5.7 |
| lag2 | 22.5±11.5 | 13.9 | 19.9 | 28.7 | 22.5±11.5 | 13.8 | 19.9 | 28.6 | 0.06±10.95 | -5.6 | 0.0 | 5.7 |
| lag0-1 | 23.1±11.0 | 14.7 | 20.9 | 29.1 | 22.8±10.9 | 14.6 | 20.6 | 28.8 | 0.23±9.74 | -4.7 | 0.2 | 5.2 |
| lag0-2 | 22.9±10.4 | 14.9 | 21.0 | 28.8 | 22.7±10.4 | 14.8 | 20.8 | 28.6 | 0.17±8.56 | -4.3 | 0.1 | 4.6 |
| CO (0.1ppm) |  |  |  |  |  |  |  |  |  |  |  |  |
| lag0 | 5.1±2.0 | 3.8 | 4.7 | 5.9 | 5.1±1.9 | 3.8 | 4.6 | 5.9 | 0.04±2.06 | -1.0 | 0.0 | 1.1 |
| lag1 | 5.1±1.9 | 3.8 | 4.6 | 5.9 | 5.1±1.9 | 3.8 | 4.6 | 5.8 | 0.02±2.03 | -1.0 | 0.0 | 1.1 |
| lag2 | 5.1±1.9 | 3.8 | 4.6 | 5.9 | 5.1±1.9 | 3.8 | 4.6 | 5.8 | 0.02±2.02 | -1.0 | 0.0 | 1.1 |
| lag0-1 | 5.1±1.8 | 3.9 | 4.7 | 5.9 | 5.1±1.8 | 3.9 | 4.7 | 5.8 | 0.03±1.82 | -0.9 | 0.0 | 0.9 |
| lag0-2 | 5.1±1.7 | 3.9 | 4.7 | 5.9 | 5.1±1.7 | 3.9 | 4.7 | 5.8 | 0.03±1.63 | -0.8 | 0.0 | 0.8 |
| O_3_ (ppb) |  |  |  |  |  |  |  |  |  |  |  |  |
| lag0 | 25.6±11.6 | 17.1 | 24.7 | 33.4 | 25.7±11.6 | 17.2 | 24.8 | 33.5 | -0.12±10.70 | -6.7 | -0.1 | 6.5 |
| lag1 | 26.2±11.8 | 17.6 | 25.3 | 33.9 | 26.2±11.8 | 17.6 | 25.3 | 34.0 | -0.01±10.75 | -6.7 | 0.0 | 6.6 |
| lag2 | 26.2±11.8 | 17.6 | 25.4 | 34.1 | 26.2±11.8 | 17.6 | 25.4 | 34.1 | 0.01±10.80 | -6.8 | 0.0 | 6.8 |
| lag0-1 | 25.9±11.1 | 17.6 | 25.0 | 33.4 | 26.0±11.0 | 17.7 | 25.1 | 33.5 | -0.07±9.31 | -5.8 | -0.1 | 5.6 |
| lag0-2 | 26.0±10.7 | 17.9 | 25.1 | 33.4 | 26.1±10.7 | 18.0 | 25.2 | 33.4 | -0.04±8.40 | -5.1 | -0.1 | 5.0 |
| PM_10_ (µg/m^3^) |  |  |  |  |  |  |  |  |  |  |  |  |
| lag0 | 48.3±27.8 | 30.8 | 43.0 | 59.2 | 48.0±27.8 | 30.5 | 42.5 | 58.5 | 0.41±36.18 | -15.9 | 0.4 | 16.9 |
| lag1 | 47.8±28.7 | 30.0 | 42.3 | 58.1 | 47.3±27.5 | 30.1 | 42.0 | 57.5 | 0.43±36.42 | -16.0 | 0.2 | 16.6 |
| lag2 | 47.7±27.9 | 30.3 | 42.1 | 58.2 | 47.2±27.1 | 30.3 | 42.1 | 57.4 | 0.48±35.69 | -16.0 | 0.3 | 16.7 |
| lag0-1 | 48.0±25.3 | 31.7 | 43.5 | 57.9 | 47.7±24.9 | 31.7 | 43.1 | 57.3 | 0.42±31.81 | -14.5 | 0.3 | 15.2 |
| lag0-2 | 47.9±23.1 | 32.7 | 44.0 | 57.3 | 47.5±22.7 | 32.7 | 43.6 | 56.6 | 0.44±28.30 | -13.0 | 0.3 | 13.8 |
| PM_2.5_ (µg/m^3^) |  |  |  |  |  |  |  |  |  |  |  |  |
| lag0 | 25.5±13.8 | 15.7 | 22.4 | 32.3 | 25.5±13.8 | 15.7 | 22.5 | 32.6 | 0.01±18.85 | -9.9 | 0.0 | 9.9 |
| lag1 | 25.2±13.6 | 15.5 | 22.7 | 31.8 | 25.3±13.7 | 15.7 | 22.8 | 32.0 | 0.07±18.51 | -9.5 | 0.0 | 10.0 |
| lag2 | 25.6±13.8 | 16.0 | 22.7 | 32.0 | 25.1±13.2 | 16.0 | 22.5 | 31.7 | 0.60±18.58 | -9.5 | 0.3 | 10.4 |
| lag0-1 | 25.3±12.3 | 16.7 | 22.8 | 31.7 | 25.4±12.3 | 16.5 | 23.1 | 32.0 | 0.04±16.47 | -8.8 | 0.0 | 8.9 |
| lag0-2 | 25.4±11.2 | 17.4 | 23.5 | 31.4 | 25.3±11.0 | 17.5 | 23.3 | 31.1 | 0.23±14.62 | -7.8 | 0.2 | 8.3 |
| Meteorological variables, lag0-3 |  |  |  |  |  |  |  |  |  |  |  |  |
| Temperature (℃) | 13.2±9.7 | 4.8 | 14.4 | 21.9 | 13.3±9.7 | 4.8 | 14.5 | 22.0 | 0.02±3.77 | -2.3 | 0.0 | 2.3 |
| Rainfall (mm) | 3.5±7.4 | 0.0 | 0.6 | 3.7 | 3.6±7.8 | 0.0 | 0.7 | 3.9 | -0.12±9.59 | -1.9 | 0.0 | 1.7 |
| Wind speed (m/s) | 2.2±0.9 | 1.6 | 2.1 | 2.7 | 2.2±0.9 | 1.6 | 2.1 | 2.7 | -0.01±0.75 | -0.4 | 0.0 | 0.4 |
| Relative humidity (%) | 66.3±12.4 | 57.9 | 67.2 | 75.6 | 66.4±12.3 | 57.9 | 67.3 | 75.8 | 0.04±11.90 | -7.7 | 0.0 | 7.7 |

**Supplementary Figure 1. Weekly concentration for each air pollutant in a typical year 2015**

**
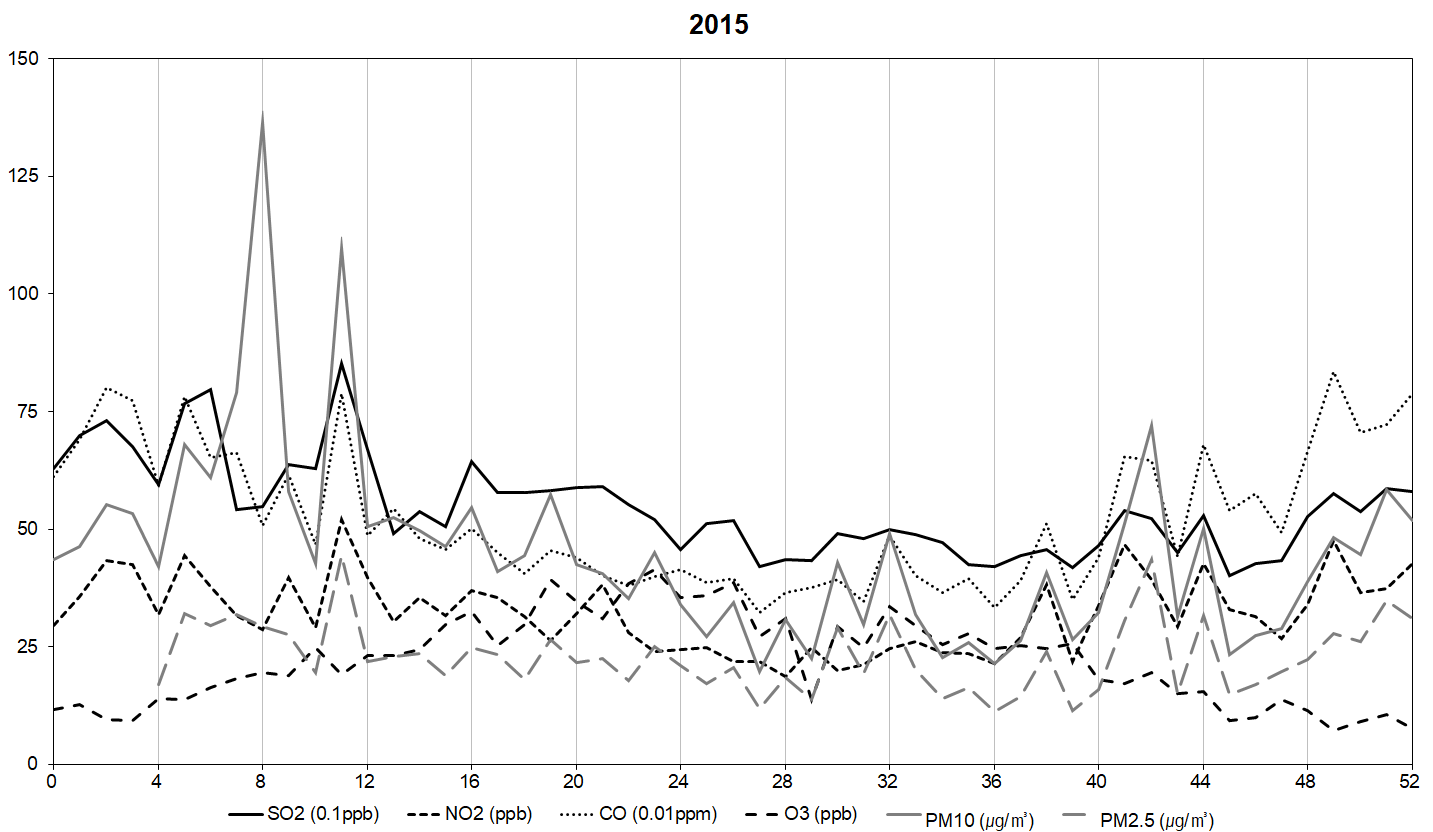
**

Weekly concentration of each air pollutant in a typical year (2015) when all pollutants were analyzed shows noticeable concentration variability between adjacent weeks for all pollutants.

*This figure was drawn using R: A Language and Environment for Statistical Computing (version 3.3.3; R Core Team, Vienna, Austria, https://www.R-project.org).*

**Supplementary Figure 2. Region-specific odd ratios (ORs) and 95% confidence intervals (CIs) and the pooled effect estimates for Meniere's disease hospital visits considering interquartile range (IQR) increase in each air pollutant concentration at lag0 (A: SO_2_; B: NO_2_; C: CO; D: O_3_; E: PM_10_; F: PM_2.5_).**

**A. SO_2_ at lag0**


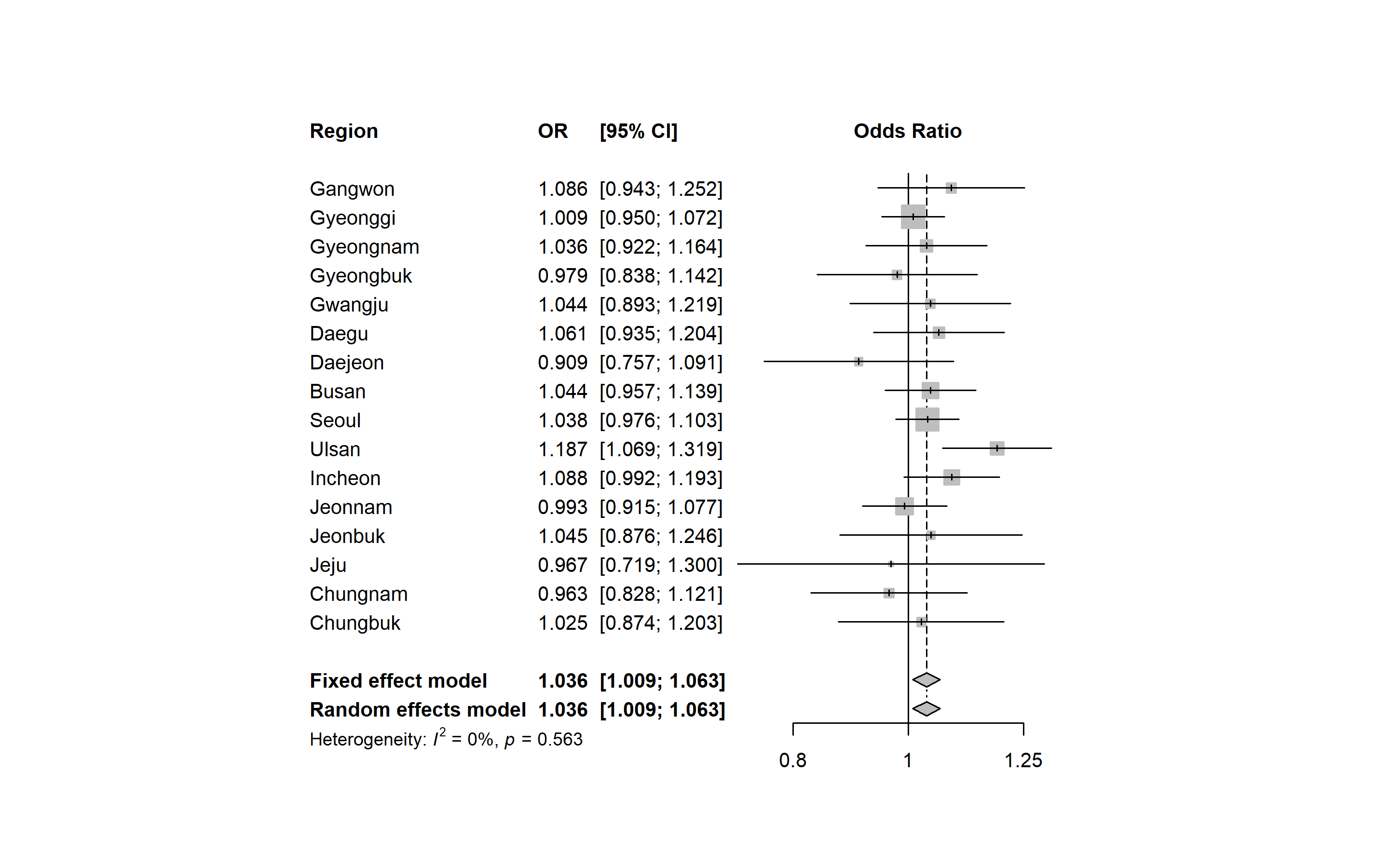


**B. NO_2_ at lag0**


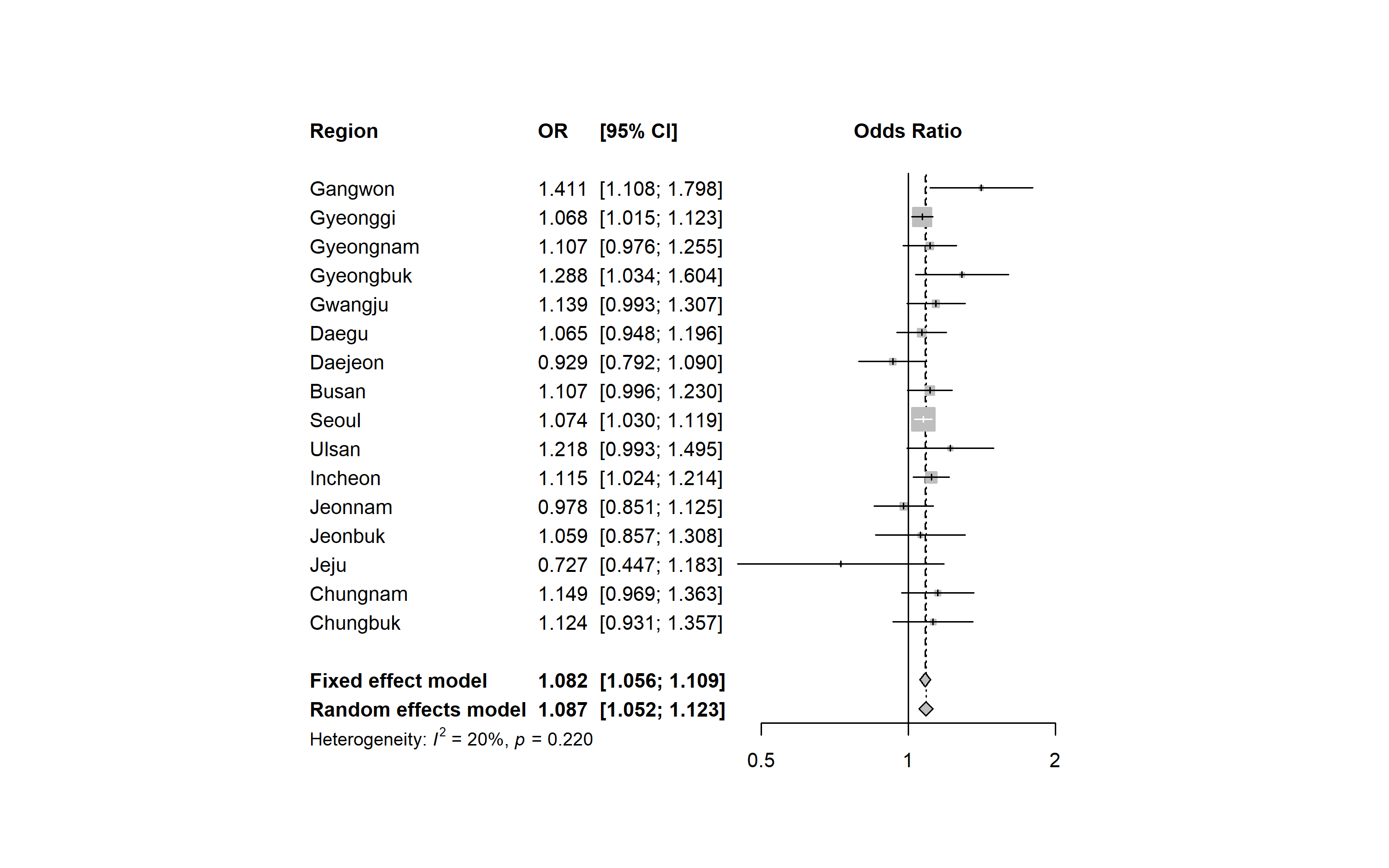


**C. CO at lag0**


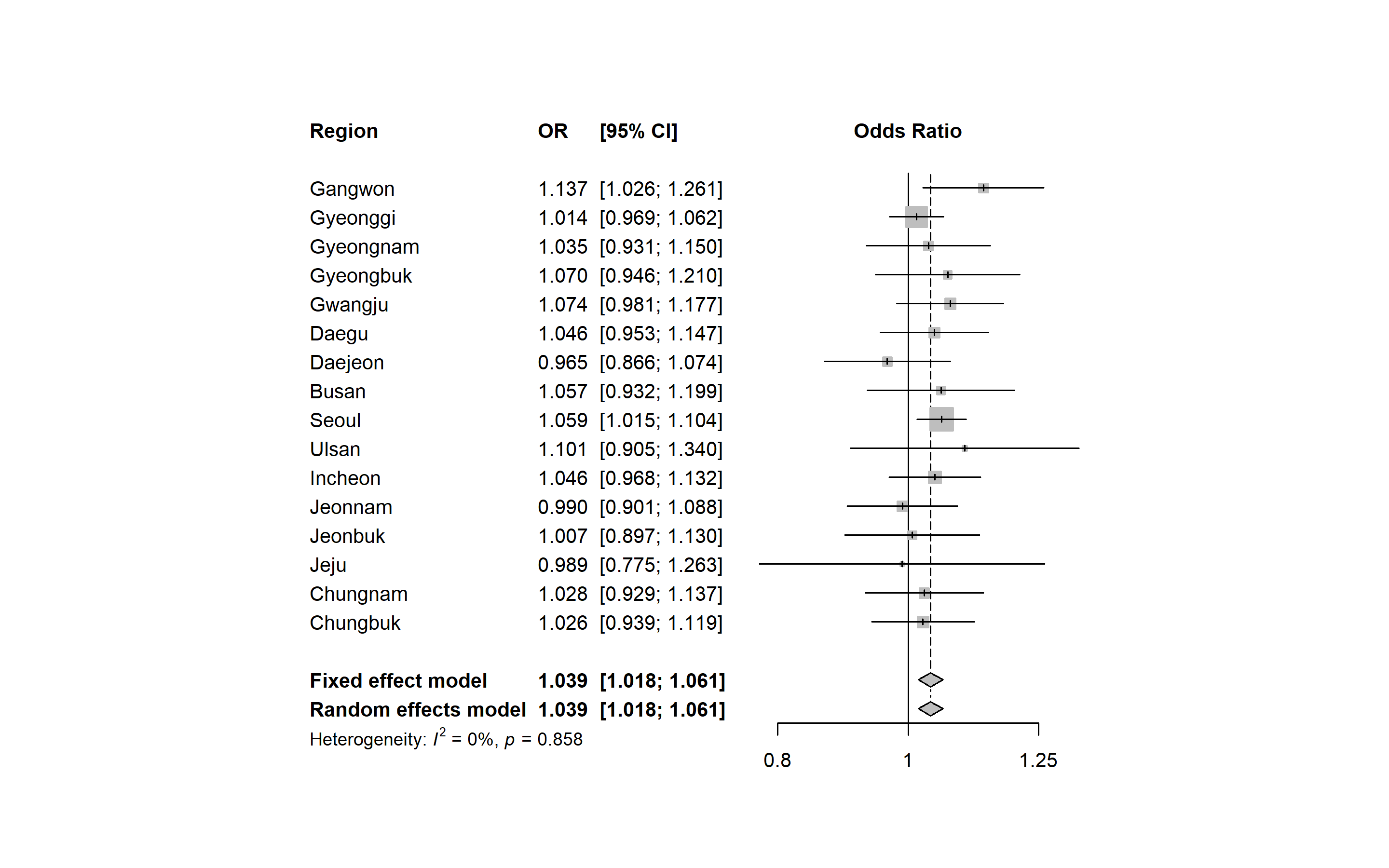


**D. O_3_ at lag0**


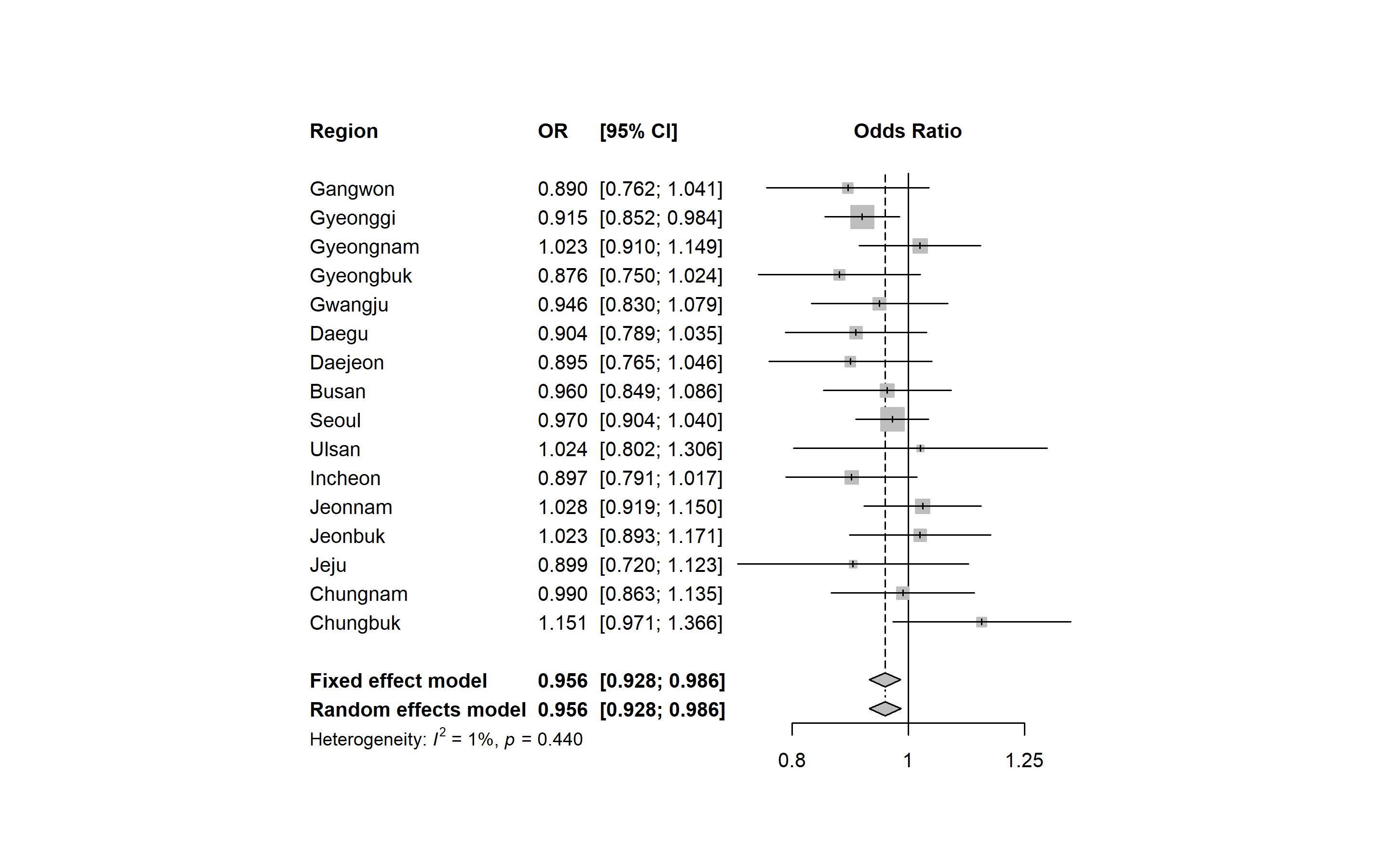


**E. PM_10_ at lag0**


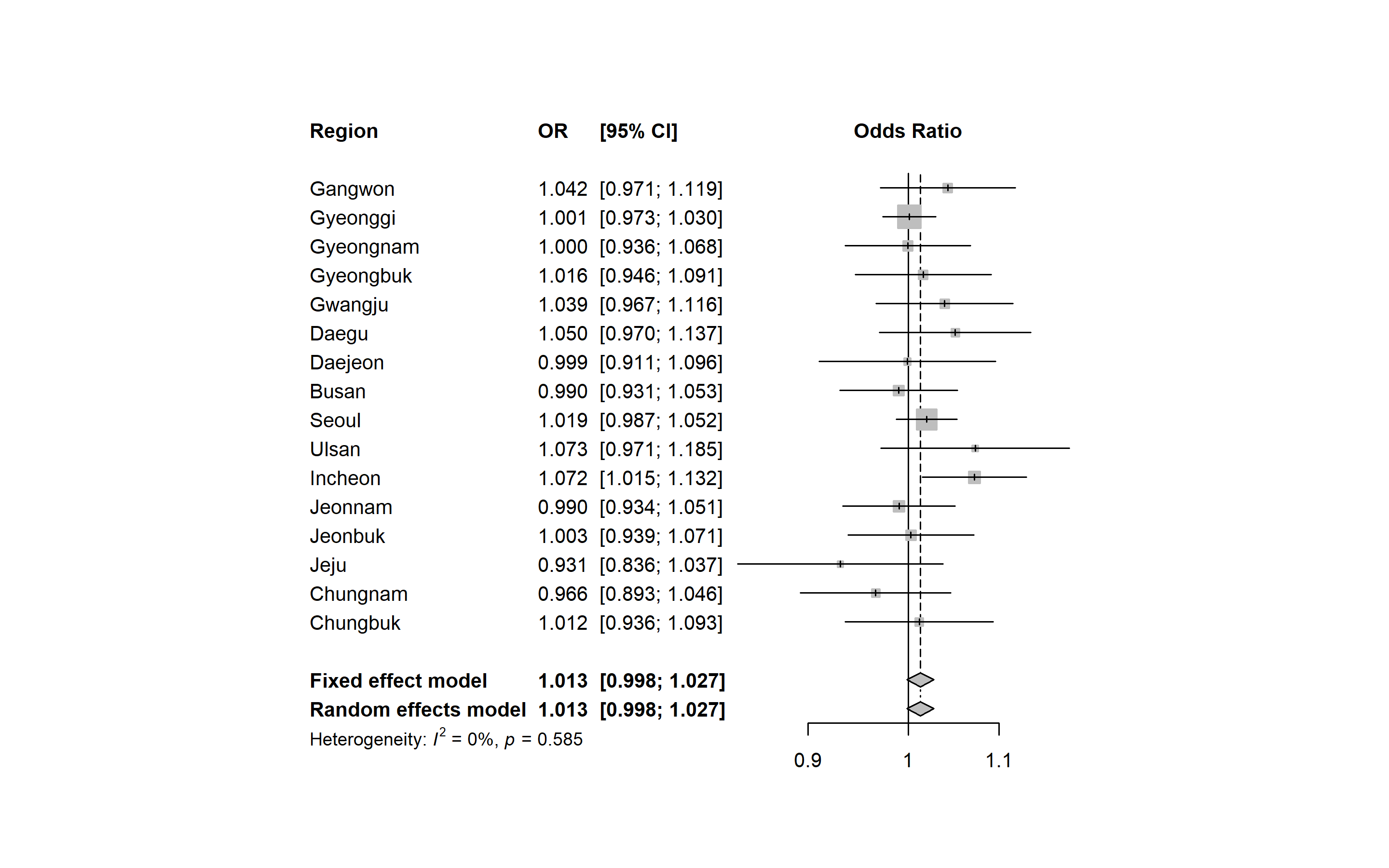


**F. PM_2.5_ at lag0**


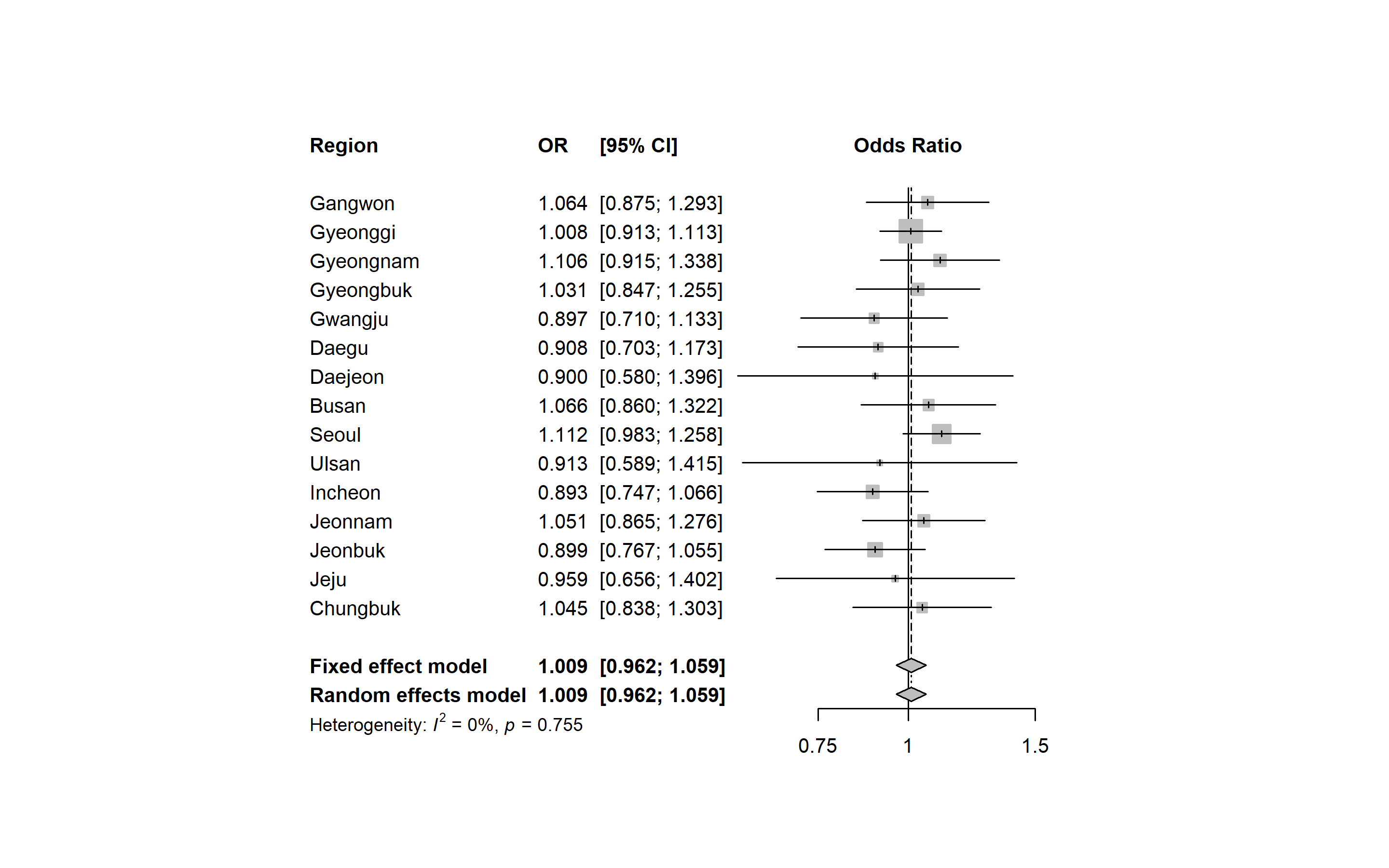


*This figure was drawn using R: A Language and Environment for Statistical Computing (version 3.3.3; R Core Team, Vienna, Austria, https://www.R-project.org).*
